# Efficacy of *Salvia officinalis* Shrub as a Sustainable Feed Additive for Reducing Ruminal Methane Production and Enhancing Fermentation in Ruminants

**DOI:** 10.3390/ani14111648

**Published:** 2024-05-31

**Authors:** Ahmed E. Kholif, Md Atikur Rahman, Salah A. H. Abo El-Nor, Tarek A. Morsy, Gouda A. Gouda, Mahmoud Fahmy, Mireille Chahine

**Affiliations:** 1 Dairy Science Department, National Research Centre, 33 Bohouth St. Dokki, Giza 12622, Egypt; nor1957@hotmail.com (S.A.H.A.E.-N.); tarekalymo@gmail.com (T.A.M.); gagouda@gmail.com (G.A.G.); fahmymahmoud2@gmail.com (M.F.); 2 Department of Animal Sciences, North Carolina Agricultural and Technical State University, Greensboro, NC 24711, USA; 3 Department of Agriculture, Nutrition and Food Systems, University of New Hampshire, Durham, NH 03824, USA; mdatikur.rahman@unh.edu; 4 Department of Animal, Veterinary and Food Sciences, University of Idaho, 315 Falls Ave., Twin Falls, ID 83301, USA

**Keywords:** in vitro fermentation, total gas production, methane, phytogenic feed

## Abstract

**Simple Summary:**

This study investigated the effect of dried and ground *Salvia officinalis* (SO) shrub leaves on ruminal fermentation characteristics, primarily focusing on nutrient degradability, in vitro gas, and ruminal CH_4_ production. Previous studies on SO mostly focused on plant extracts or sage essential oil with different inclusions. This study used dried and ground leaves of SO in an in vitro experiment to evaluate the dose response when the ruminants’ diets were supplemented with incremental amounts of SO. We found that a 1% inclusion of SO significantly improved gas production kinetics, nutrient degradability, and the ruminal fermentation profile while reducing CH_4_ production significantly. This study contributes to developing our understanding of phytogenic feed additives and their potential use in ruminants’ diet as sustainable method to reduce the environmental impact of livestock farming.

**Abstract:**

The objective of this study was to evaluate the effects of the inclusion of dried *Salvia officinalis* (SO) shrub leaves on nutrient degradability, ruminal in vitro fermentation, gas production (GP), methane (CH_4_), and carbon dioxide (CO_2_) productions. Dried and ground SO shrub leaves were included at 0% (control), 0.5%, 1%, 1.5%, and 2% DM of a diet consisting of (per kg DM) 500 g concentrate feed mixture, 400 g berseem hay, and 100 g rice straw. The diet was incubated for 48 h. The asymptotic GP and the rate of GP changed linearly and quadratically (*p* < 0.01), with the highest GP observed at 1% inclusion of SO and then decreasing thereafter with greater inclusion (i.e., 1.5% and 2%), while CH_4_ production and its rate decreased linearly (*p* < 0.01) with all levels of SO inclusion. A linear increase in CO_2_ production and its rate was also found with an increasing level of SO inclusion in the diet (*p* < 0.05). Furthermore, the degradability of DM, NDF, and the concentration of total short-chain fatty acids and acetate changed linearly and quadratically, with the greatest being found at 1% SO inclusion and then steadily declining after (*p* < 0.01) with the 1.5% and 2% inclusion levels. Meanwhile, the propionate, NH_3_-N, and microbial crude protein levels showed similar trends, with the plateau found at 1% inclusion of SO, where there was no change in butyrate concentration. Moreover, the pH, metabolizable energy, and partitioning factor (PF_24_) also changed linearly and quadratically (*p* < 0.05), where the pH and PF_24_ were considerably reduced and ME increased with a 1% inclusion of SO (*p* < 0.05). In summary, SO at 1% inclusion in the diet showed the potential to improve gas production kinetics, nutrient degradability, and the ruminal fermentation profile, with a more significant reduction in ruminal CH_4_ production suggesting that SO at 1% could be included in the ruminant diet to reduce their carbon footprint and increase the production performance.

## 1. Introduction

Greenhouse gas emissions are a considerable environmental concern,, and it is estimated that 14.5% of global anthropogenic gas emissions are contributed by livestock [1]. Methane (CH_4_), a particularly very potent greenhouse gas that is 28 times more powerful than carbon dioxide (CO_2_) when it comes to trapping heat in the atmosphere over a 100-year period [2]. According to the EPA, 25% of the total CH_4_ emissions in the US originate from ruminant animals (i.e., cattle, goats, and sheep). In addition to environmental concerns, CH_4_ production makes animals less energy efficient, with10% of energy being lost through CH_4_ production [3]. Of the greenhouse gases emitted from enteric fermentation, 71% come from ruminal fermentation [4]. Thus, it is essential to prioritize CH_4_ mitigation research that focuses on ruminal fermentation. Intensive research has been carried out in the past decade with a significant focus on finding anti-methanogenic feed additives (e.g., plant secondary metabolites, enzymes, ionophores, and seaweeds) to reduce enteric CH_4_ emissions [5,6,7,8]. However, very few nutritional-based strategies were effective in vitro and in vivo and generally recognized as safe compounds by the US Food and Drug Administration.

The *Salvia officinalis* shrub, commonly known as sage, is rich in polyphenols, rosmarinic acid, camphor, and carnasol and has been shown to reduce the molar concentration of butyrate and CH_4_ production while increasing the acetate concentration, total gas production, and volatile fatty acids concentrations in vitro [9]. Similarly, a recent study [10] compared the nutritional value of sage, adiantum, and alfalfa and their different inclusion rates in the diet of ruminants and reported that sage did not have any effect on in vitro pH, gas production, total volatile fatty acids concentration, organic matter digestibility, or metabolizable energy. However, it increased the substrate’s true degradability while decreasing the protozoa population, methane and ammonia production, and acetate to a propionate ratio [11]. It has also been found that gas production was lower with the higher doses of sage in vitro. Interestingly, inclusion of sage essential oil at different doses did not affect ruminal methane production in a previous study [12]. Generally, the plant secondary metabolites in many herbal plant extracts have been shown to be rumen modifiers [13], suggesting that they have potential antimicrobial activities against a wide range of bacteria, yeasts, and molds [14,15,16].

There has been an increasing amount of effort aimed towards finding alternative feedstuffs for the formulation of next-generation animal feed that has less issues regarding its safety and efficacy, is scalable, and enhances the production performance of the animals and overall sustainability of the farm. Phytogenic feed additives can serve as a substitute for synthetic feed additives to enhance the environmental sustainability of livestock farming, which may be more appealing to consumers. The investigation of feeding techniques and the use of feed additives to lessen the environmental impact of ruminant production is being extensively studied due to its effectiveness in increasing animal efficiency while minimizing nutrient excretion.

To date, only a few studies have investigated the suitability of using SO as animal feed [9,10,12,17,18]. Also, the concentrations of secondary metabolites are much higher if whole plants are used rather than just leaves, which could be detrimental to rumen health and negatively affect ruminal fermentation parameters [16,19]. Hence, this study aimed to evaluate the effects of incorporating different doses of dry SO shrub at 0.5%, 1%, 1.5%, and 2% concentrations in a total mixed ration on in vitro gas, CH_4_, and CO_2_ production, and the ruminal fermentation profile. Our hypothesis was that the phytochemicals present in the dried SO shrub would have an impact on ruminal fermentation, enhance the degradability of nutrients, and reduce CH_4_ production in vitro.

## 2. Materials and Methods

### 2.1. Ingredients and Treatments

A basal total mixed ration (TMR) was formulated as a substrate, containing the following ingredients per kg of dry matter (DM): 500 g of concentrate feed mixture, 400 g of berseem hay, and 100 g of rice straw. The nutrient compositions of SO shrubs and TMR are listed in Table 1.

Clean and dry SO shrub was obtained from a local supplier in Egypt. S. officinalis shrub was grounded and mixed before use. The essential oils in the shrub were measured at the Central Laboratory of National Research Centre (Egypt) using a Perkin Elmer Auto System XL GC/MS (Agilent, Santa Clara, CA, USA), and a capillary column ZB-5 (60 m × 0.32 mm i.d.; Agilent, USA). The injector temperature was set at 50 °C for 1 min, then programmed to 240 °C at 3 °C/min. Helium was used as the carrier gas at the rate of 1 mL/min with a split vent flow of 1:10. The effluent in the GC column was introduced directly to the source of the MS. Spectra were obtained in the EI mode with 70 eV ionization energy. The sector mass analyzer was set to scan from 40 to 300 amu for 1s. A tentative identification of the compounds was performed based on the comparison of their relative retention time and mass spectra with those of the NIST and WILLY library data from the GC-MS system.

### 2.2. In Vitro Fermentation and Biodegradation

The in vitro fermentation medium was prepared according to Goering and Van Soest [20]. A reducing solution containing sodium sulfide (2 mL) was added to the buffer shortly before the addition of rumen fluid. Ruminal inoculum (20 mL) and the buffer solution (80 mL) were mixed in a 250 mL bottle.

Ruminal inoculum was collected from the rumen of three sheep from a local slaughterhouse in Cairo (Egypt). Before slaughtering, sheep were ad libitum fed a diet containing concentrates, berseem hay, and rice straw at 500:400:100 (DM basis), with free access to water. The slaughter of the animals was carried out following the animal welfare regulations. The rumen contents were collected considering the standardized procedure for sampling, storage, and use of ruminal contents recommended by Fortina et al. [21]. All required consent for the procurement of fluid from slaughtered animals for research purposes was obtained from the slaughterhouse. At the slaughterhouse, the interval between the animal death and the rumen fluid collection was less than 10 min. About 150–250 g of rumen content was sampled by hand and squeezed into a plastic beaker, using the colander; then, the process was repeated until approximately 1000 mL of rumen fluid was collected. The collected fluid was filtered through two-layers of cheesecloth to remove large feed particles, and the particulate materials were squeezed to obtain microbes attached to the feed particles. The initial pH of the inoculum ranged from 6.8 to 6.9. All treatments were tested in two incubation runs with 3 replicates in each run. In each incubation run, 2 bottles filled with inoculum but without feed (blanks) were also included to establish baseline fermentation GP. All the incubations were performed together at the same time.

A 1 g ± 10 mg sample for TMR was weighed into filter bags (ANKOM F57; Ankom Technology, Macedon, NY, USA) and then placed into 250 mL ANKOM bottles (Ankom^RF^ Gas Production System) fitted with an automatic wireless in vitro GP module (Ankom Technology, Macedon, NY, USA) with pressure sensors. The *S. officinalis* shrub was included in the diet at different levels: 0% (control), 0.5%, 1%, 1.5%, and 2%. Pressure measurements were recorded at 10 min intervals for a duration of 48 h, and the cumulative pressure was calculated based on these measurements. The gas pressure data were subsequently converted into gas volume (measured in mL) using standard temperature and pressure conditions. Net gas output was determined by subtracting the gas volume of the blank samples. During each incubation time point (i.e., 2, 4, 6, 8, 10, 12, 24, 36, and 48 h of incubation), 5 mL gas samples were collected from the sampling vent and analyzed using a Gas-Pro detector (Gas Analyzer CROWCON Model Tetra3, Abingdon, UK) to measure the concentrations of CH_4_ and CO_2_.

### 2.3. Sampling and Analysis of Fermentation Variables

Following a 48-h incubation period, the fermentation process was halted by placing the bottles on ice for a duration of 5 min. Subsequently, the pH level was promptly measured using a pH meter. The ANKOM F57 filter bags were dried in a forced air oven at a temperature of 55 °C for 48 h. The degradation of dry matter (*d*DM), NDF (*d*NDF), and acid detergent fiber (*d*ADF) was determined by subtracting the weight of the dried residue from the initial weight of the dried substrate. Total gas, CO_2_, and CH_4_ productions were quantified relative to the changes in *d*DM, *d*NDF, and *d*ADF after 48 h of incubation.

Approximately 5 mL samples of the fermented fluid supernatant from each bottle were collected in glass tubes for the purpose of determining ammonia-N (NH_3_-N) levels and total and individual short-chain fatty acids (SCFAs) concentrations. For the quantification of NH_3_-N concentration according to AOAC, a 3 mL subsample was treated with 3 mL of a 0.2 M hydrochloric acid solution. An aliquot (0.8 mL) was combined with 0.2 mL of a solution containing metaphosphoric acid (250 g/L) for SCFAs analysis.

### 2.4. Chemical Analysis

The ash content of *S. officinalis* shrub and TMR samples was determined by burning the samples in a muffle furnace at 550 °C for 12 h (method ID 942.05). The protein content (CP) was analyzed using the Kjeldahl method (method ID 954.01), and the ether extract (EE) was determined using diethyl ether in Soxhlet extractors (method ID 920.39) following the AOAC [22] methods. The neutral detergent fiber content was measured using sodium sulfite, following the procedure outlined by Van Soest et al. [23], without the use of alpha amylase. The acid detergent fiber content was determined using the AOAC [22] technique (ID 973.18) and reported without including any remaining ash. Concentrations of non-structural carbohydrate, cellulose, hemicellulose, and organic matter (OM) were determined.

### 2.5. Calculations and Statistical Analyses

The NLIN procedure for SAS (version 9.4, SAS Inst., Inc., Cary, NC, USA) was used to fit the total GP (mL/g DM) data for the calculation of GP kinetics. The model used was based on France et al.’s [24] and may be represented as: y = A × [1 − e^−c (t−Lag)^] The equation represents the relationship between the volume of total GP production at time t (h), denoted as y, and the variables A, c, and Lag (h). A is the asymptotic GP (mL/g DM), c represents the fractional rate of GP (/h), and Lag (h) indicates the discrete lag time before any GP.

The partitioning factor at 24 h of incubation (PF_24_; mg dDM: mL gas) was estimated [25]. The volume of gas produced (mL/200 mg DM) at 24 h incubation (GY_24_) was calculated. Metabolizable energy (ME) was calculated according to Menke et al. [26]. Microbial crude protein (MCP) production was calculated according to Blümmel et al. [25].

The data were analyzed using the Generalized Linear Model (GLM) process of SAS in a completely randomized design. The following model was used: Y_ij_ = μ + L_i_ + ε_ij_, where Y_ij_ is the observation, μ is the population mean, L_i_ is the effect of incremental inclusion of *S. officinalis*, and ε_ij_ is the residual error. Linear and quadratic contrasts were used to determine the level responses (increasing *S. officinalis* levels).

## 3. Results

### 3.1. Salvia Officinalis

The volatile compounds that were found in the SO are shown in Table 2. The concentrations of the major volatile compounds identified in SO were as follows: α-pinene (15.85%), α-thujone (15.62%), camphor (15.20%), 1, 8-cineole (13.20%), and β-pinene (10.84%). Additionally, some of the minor volatile compounds found in SO were camphene (6.86%), limonene (5.22%), β-thujone (3.90%), borneol (2.30%), β-caryophyllene (2.20%), *p*-cymene (2.0%), and β-myrcene (1.52%). There were also some other volatile compounds identified with a lower concentration, such as α-terpineol (0.76%), thujone (0.50%), verbenol (0.37%), L-fenchone (0.35%), estragole (0.31%), linalool (0.28%), and tricyclene (0.17%).

### 3.2. Biogases Production

**Figure 1:**
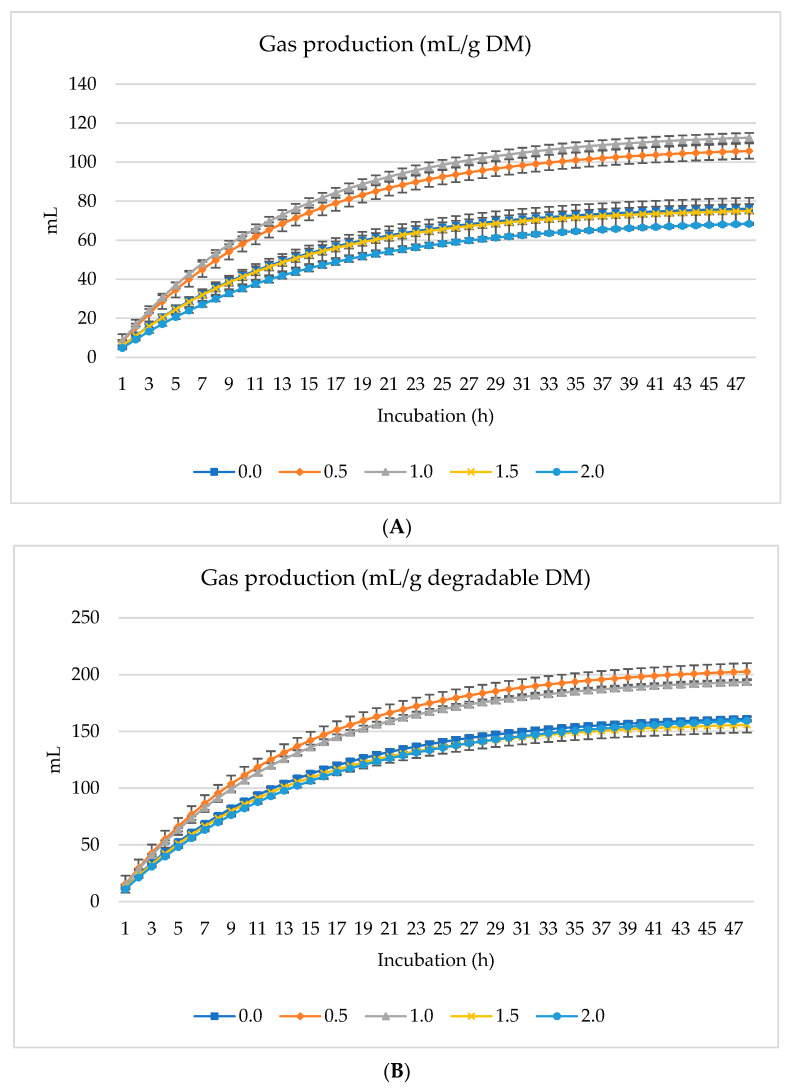

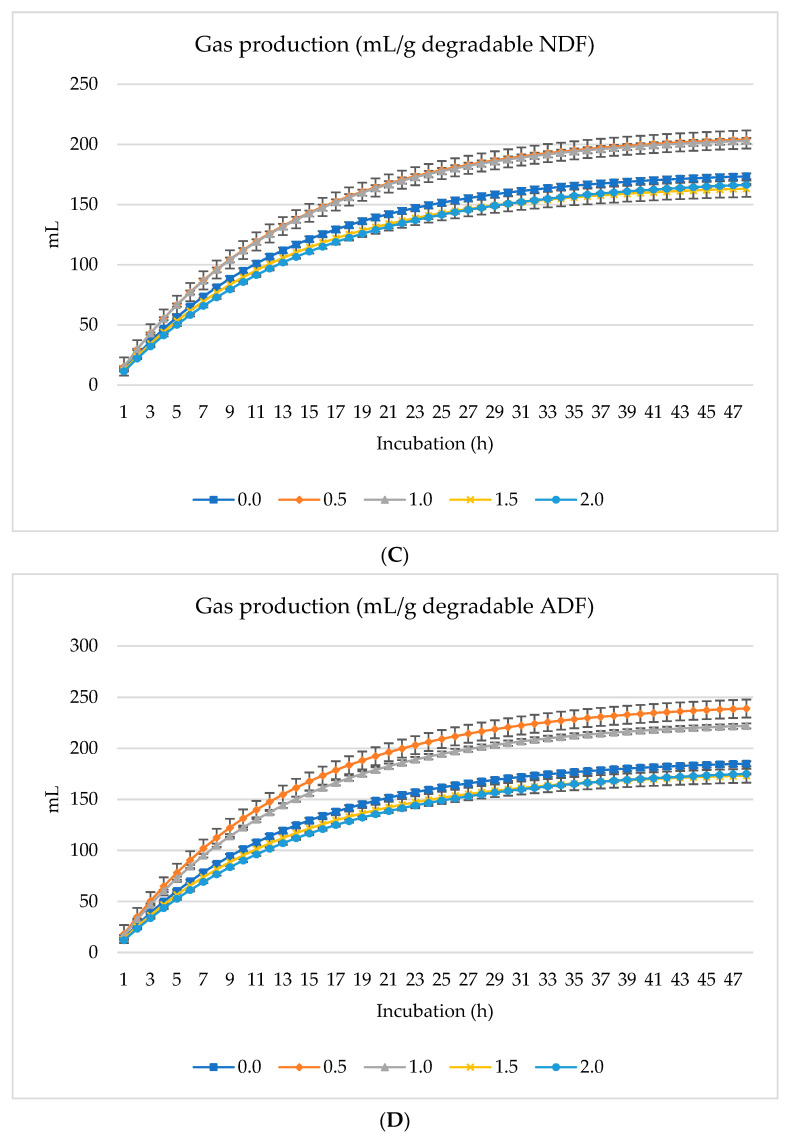
In vitro ruminal gas production: mL/g incubated DM (**A**), mL/g degradable DM (**B**), mL/g degradable NDF (**C**), mL/g degradable ADF (**D**) of a total mixed ration supplemented with different levels of *Salvia officinalis* shrub.

**Figure 2:**
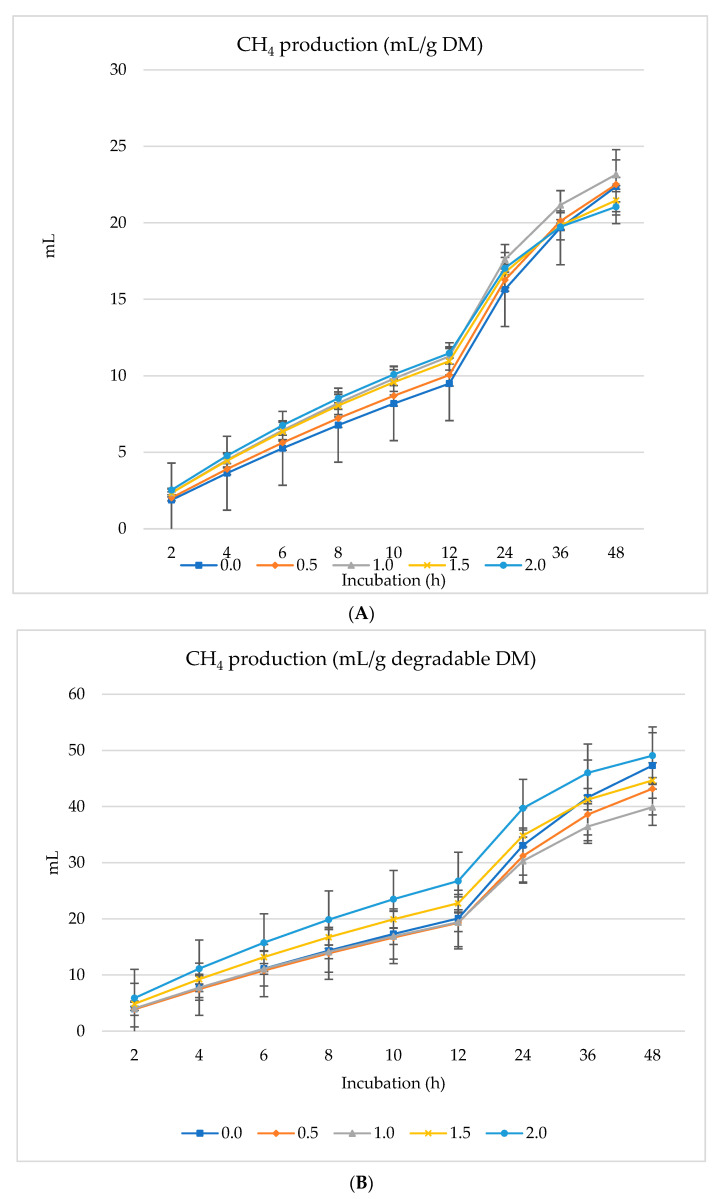

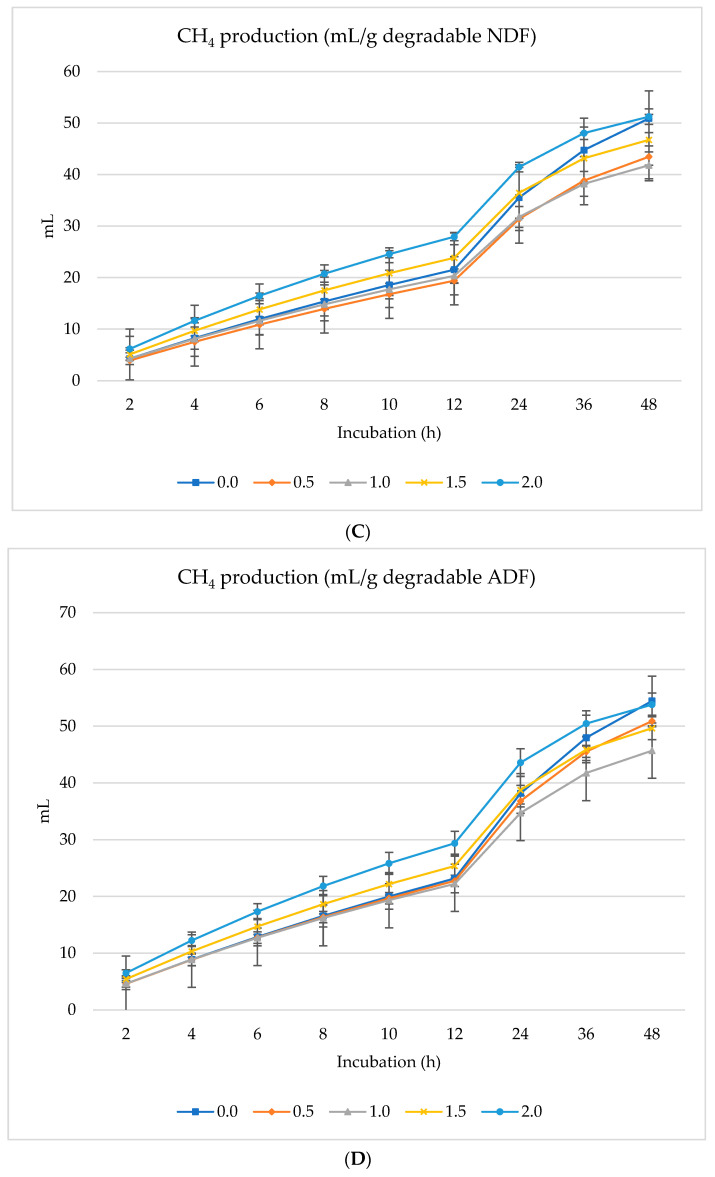
In vitro CH_4_ production: mL/g incubated DM (**A**), mL/g degradable DM (**B**), mL/g degradable NDF (**C**), mL/g degradable ADF (**D**) of a total mixed ration supplemented with different levels of *Salvia officinalis* shrub.

**Figure 3:**
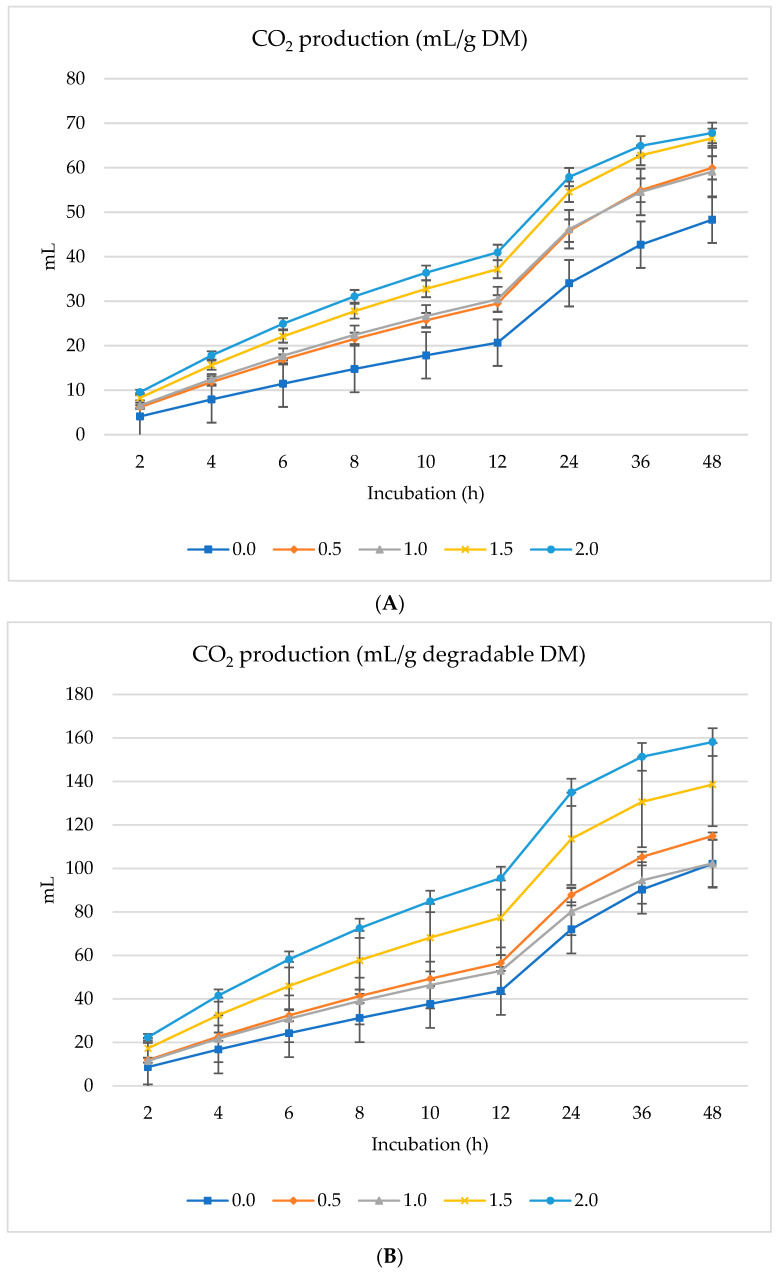

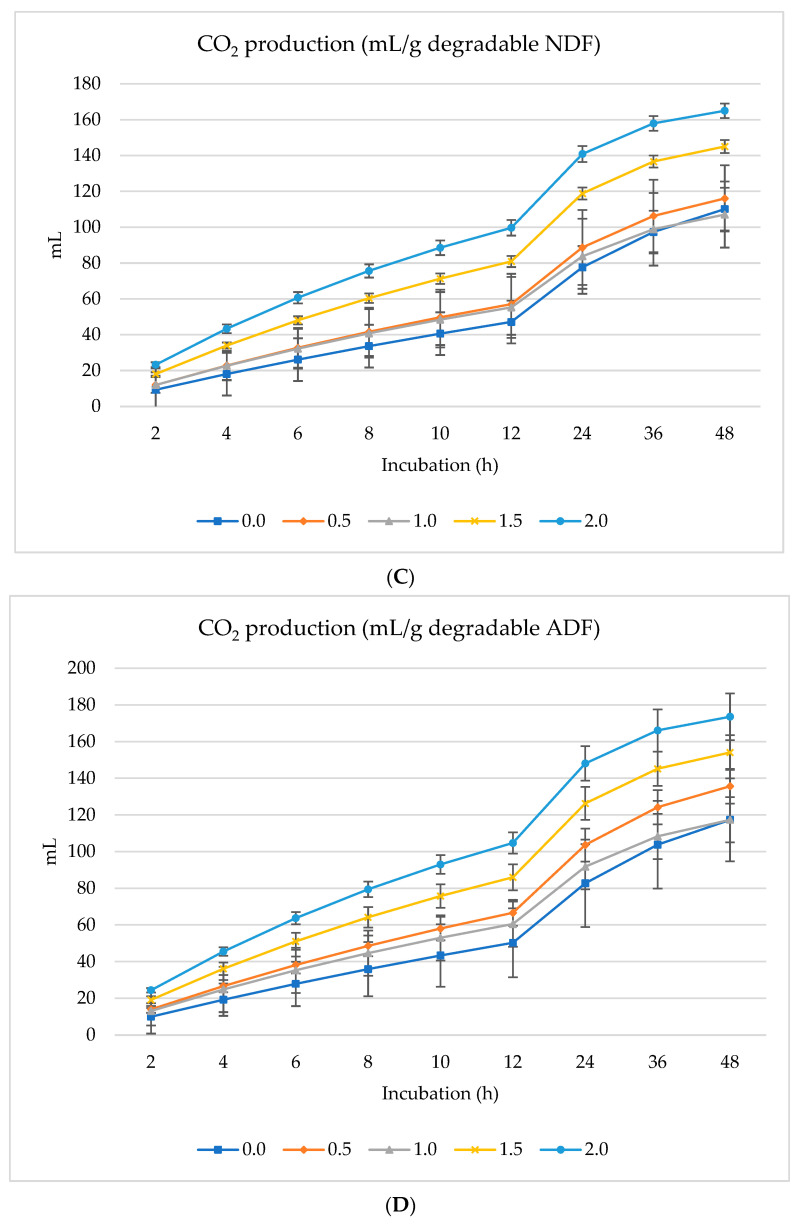
In vitro CO_2_ production: mL/g incubated DM (**A**), mL/g degradable DM (**B**), mL/g degradable NDF (**C**), mL/g degradable ADF (**D**) of a total mixed ration supplemented with different levels of *Salvia officinalis* shrub.

Figure 1, Figure 2 and Figure 3 show GP (mL), CH_4_ (mL), and CO_2_ (mL) per g DM, *d*DM, *d*NDF, and *d*ADF. Gas production (mL/g DM) increased linearly with the incubation time (h) where increased gas production (mL) was overserved with 1% inclusion of SO followed by 0.5% dietary inclusion (Figure 1A). The lowest gas production was found when 2.0% SO was included in the diet (Figure 1A). In contrast, 0.5% SO inclusion had the optimum gas production per g of degradable DM (Figure 1B). Similarly, the gas production was higher and increased linearly with 0.5% SO inclusion when considered per g of degradable NDF and ADF (Figure 1C,D). Ruminal CH_4_ and CO_2_ production also increased linearly with the increasing amounts of SO in the diet; however, CH_4_ and CO_2_ production peaked with 2.0% dietary inclusion of SO per g of DM, degradable DM, ADF, and NDF (Figure 2 and Figure 3). The inclusion of incremental amounts of SO changed the GP (mL/g DM) linearly (*p* < 0.001) and quadratically (*p* < 0.001) after 48 h of incubation, increasing from 0% to 1% and then drastically decreasing from 1.5% to 2% (Of total DM in the diet). Similarly, the rate of GP changed linearly (*p* = 0.01) and quadratically (*p* = 0.01), increasing from 0% to 1% and then the rate of increase gradually diminished and decreased with the addition of 1.5% or 2% of SO to the diet (Table 3). Asymptotic CH_4_ production (mL/g DM) decreased linearly (*p* < 0.01) with incremental levels of SO in the diet. Contrarily, the rate of CH_4_ production (/h) increased linearly (*p* = 0.006) with the inclusion of SO while the lag phase of CH_4_ production was also extended linearly (*p* < 0.001). The production of asymptotic CO_2_ (mL/g DM) changed linearly (*p* = 0.005), increasing as the inclusion level of SO progressed. Similarly, the rate of CO_2_ production (/h) also increased linearly (*p* = 0.002).

### 3.3. Degradability and Fermentation

The inclusion of SO changed the *d*DM and *d*NDF linearly (*p* = 0.004) and quadratically (*p* < 0.001), increasing with incremental levels of SO in the diet and reaching a plateau at 1% of SO and then declining after that with 1.5% and 2% inclusion (Table 4). The *d*ADF exhibited a quadratic (*p* = 0.004) change, with the highest degradation observed at 1% inclusion and then decreasing afterward. The total SCFA changed both linearly (*p* < 0.001) and quadratically (*p* < 0.001), with the highest concentration (mmol/g DM) observed at 1% inclusion of SO, and then steadily declining from 1.5% to 2%. Likewise, the acetate concentrations (mmol/g DM) changed linearly (*p* = 0.002), and quadratically (*p* < 0.001), increasing from 0% to 1% and then declining afterward, with the lowest concentration observed at 2% inclusion of SO. Whereas a quadratic response (*p* = 0.008) was observed for propionate concentrations (mmol/g DM), peaking at 1% and then decreasing in a similar trend, there was no effect (*p* = 0.121) of different levels of SO on the butyrate concentration. The fermentation pH was changed linearly (*p* = 0.018) and quadratically (*p* = 0.002), decreasing from 0 to 1% and then increasing with the highest inclusion of SO. The treatments did not affect the concentration of NH_3_-N (mg/g DM). Metabolizable energy (ME; MJ/kg DM; linearly at *p* < 0.001 and quadratically at *p* < 0.001) and PF_24_ (linearly at *p* = 0.003 and quadratically at *p* = 0.001), and microbial crude protein (MCP; linearly at *p* = 0.004 and quadratically at *p* = 0.002) changed when ME and MCP reached their peak at 1% and then followed a downward pattern up to 2% inclusion. However, PF_24_ decreased from 0% to 1% before increasing sharply with the 2% inclusion.

## 4. Discussion

### 4.1. Salvia Officinalis

Three major volatile compounds found in the SO were α-pinene (15.85%), α-thujone (15.62%), and camphor (15.20%). Previous research evaluating different extraction methods on the yield of polyphenolic and volatile compounds of SO from leaves and stem indicated that the oil content from the leaves and stem was similar, but the concentration of thujone was higher in the stem than the leaves, which is considered to be toxic for the brain, kidney, and liver if a large amount is consumed [27]. However, dried SO leaves were used in the current study, offering a safe and high-quality option. Additionally, the concentration of volatile compounds found herein was consistent with Sharma et al. [27]. The nutritional and chemical composition of SO depends on various factors (fresh vs. dried, vegetative growth, drying temperature, oven drying vs. freeze drying, harvesting time, and maturity). Baranauskiene et al. [28] reported that the concentration of volatile compounds in dried samples was 1.3–1.8 times lower than that in fresh samples when dried at 40 °C in the dark [28]. If harvested between May and June, the number of volatile compounds steadily increased due to the rainfall regime in this period. However, the discrepancies in the chemical composition of SO originate from multiple sources. For instance, variations in geographical locations, phenological stage, and the agronomic conditions of the plant sources, as well as changes in the storage and extraction methods used, could lead to a different concentration of volatile compounds [28].

These volatile compounds from plants have numerous pharmacological properties, including antimicrobial, anti-inflammatory, and antioxidant [29]. The intervention in ruminal fermentation by changing specific ingredients in the diet has emerged as a practical approach to modulating rumen digestion kinetics, reducing the carbon footprint of ruminant animals, and increasing the production efficiency of animals, considering its crucial role in digestive physiology, nutrient metabolism, and altering the rumen microbiome. Various dietary interventions offer avenues for rumen microbiome modulation, with plant volatile compounds potentially modulating and manipulating rumen fermentation due to their potential effect on ruminal microorganisms [30]. For example, oxygenated terpenoids have been shown to strongly inhibit the growth and activity of rumen microorganisms [11]. Moreover, these compounds also act as antimicrobial agents against bacteria, protozoa, and fungi, which includes the perturbation of cell membrane integrity, the modulation of signal transduction pathways, the inhibition of enzymatic activity, and interference in bacteria colonization [29]. Rumen kinetics and gaseous production responded differently in previous in vitro experiments with sage essential oil from plant extracts and dried leaves of SO [17]. The difference indicates that the quantity of volatile compounds differs between the two forms of SO, resulting in distinct mechanisms of action on ruminal microbes and consequent modifications to the ruminal fermentation profile.

### 4.2. Gas Production

Gas production kinetics provide a valuable insight into the digestibility of the feedstuff, ruminal fermentation, and microorganisms, which aids in the assessment of the impact of any feed additive on ruminal microbes [31,32]. Therefore, it can contribute to advancing knowledge regarding the microbial response to any feed additive if exposed for a more extended period of time [33]. In the current study, incremental levels of SO changed the asymptotic gas production linearly and quadratically, increasing with the inclusion of 0% to 1% SO in the diet and then decreasing from 1.5% to 2% of SO in the diet DM, suggesting a dose-dependent effect of SO on cumulative gas production. Previously, only a few studies had evaluated the impact of SO on ruminal fermentation and gas production in vitro. While there were no changes in gas production parameters in a previous study with 15% or 30% of SO in the diet DM [10], Zmora et al. [9] observed greater total gas production with increasing levels of SO (0, 4.0, 10.0, 20.0, 40.0, 100.0, and 200.0 mg/incubation vessel); however, the lowest total gas production was found with the highest level of SO supplementation which is in agreement with the findings of the current study [12]. Our results for asymptotic gas production are also consistent with Kahvand et al. [12] in which gas production was greater with a low dose (250 mg/L) and decreased thereafter with higher levels (500 mg/L and 750 mg/L) [12]. The primary mechanism through which essential oils from various plant extracts can affect rumen fermentation and gas production is through their antimicrobial properties and the effectiveness of these properties depends on the dosage and concentration of bioactive compounds, which can vary due to the nature of the products [34,35]. Considering the specific chemical composition of certain feed additives, higher doses may have certain inhibitory effects on a wide range of microorganisms in the rumen, thereby changing the gas production characteristics. In particular, the decreased gas production in the current study with the greater inclusion could be explained by the strong inhibitory effects of the volatile compounds (i.e., α-pinene, α-thujone, and camphor) within SO on a wide range of microorganisms in the rumen and the greater *d*DM and *d*NDF also explain the highest GP with 1% inclusion compared to the other levels. Additionally, there is evidence indicating that certain components found in essential oils from plant extracts, particularly those with lower antimicrobial potential, like monoterpenoids with hydrocarbon and alcohol structures, could potentially act as a carbon source for certain microorganisms in the rumen [17]. The chemical composition of sage, being composed of greater amounts of α-pinene, α-thujone which are the sources of monoterpene hydrocarbons, may potentially change the rumen fermentation and gas production parameters.

Furthermore, whereas the dose-dependent response of the rate of gas production (/h) increased linearly with incremental levels of sage essential oil [12,18], a linear and quadratic response was observed in the current study, with the rate of gas production peaking at 1% inclusion of SO, and then decreasing thereafter. The lag in GP followed a similar trend, which confirms the inverse relationship between the lag in GP and asymptotic gas production [36,37], indicating that the highest GP with minimal lag was observed at 1% inclusion of SO. Moreover, the longer lag time (L) observed when using 1.5% and 2.0% SO inclusions may be due to the inhibitory effects of certain chemicals and the subsequent period of time needed for microbes to adjust to the SO, resulting in a delayed beginning of fermentation and gas production.

### 4.3. Methane and Carbon Dioxide Production

It was expected that the inclusion of SO would show anti-methanogenic activity by improving substrate degradability and the ruminal fermentation profile, and inhibiting methanogenic archaea, thereby decreasing CH_4_ production. The production of CH_4_ decreased linearly in the current study, which is concurrent with the findings of others in which dried leaves and sage essential oil were evaluated in vitro [9,10,18,38]. Patra and Saxena [39] demonstrated that essential oils, organosulfur compounds, and flavonoids can impact CH_4_ production through the direct inhibition of methanogens without altering the population of protozoa. The study conducted by Broudiscou et al. [17] provided evidence that extracts derived from SO are abundant in flavonoids and exhibit efficacy in mitigating methanogenesis in ruminal microorganisms within a continuous culture system while not affecting the populations of protozoa [17]. The linear decrease in CH_4_ production may also be explained by the total SCFA concentration and the molar proportion of individual SCFA, where the propionate concentration increased from 0 to 1% of SO inclusion and then decreased with the highest dose at 2% SO inclusion. It has been suggested that an increasing amount of propionate can act as a hydrogen sink and can limit the availability of CH_4_ production precursors (CO_2_, H_2_) by shifting the [H^+^] from the formation of CH_4_, thereby decreasing methanogenesis [40,41]. However, acetate and butyrate followed the same trend, changing linearly and quadratically. Therefore, the linear decrease in CH_4_ may not be solely due to the alterations in SCFA production. Instead, it is likely that this reduction is a consequence of the direct suppression of methanogens. Previous studies have demonstrated that plant secondary compounds can influence protozoa populations, interfere with methanogens and hydrogen-consuming bacteria, and modify their metabolic processes [9,10].

Indeed, the negative effects of these phytogenic feed additives are highly dose-dependent, as shown by others. In contrast to our findings, other researchers did not find any difference in CH_4_ production when comparing different doses of sage oil vs. a control diet in vitro [12,42]. The discrepancies in the findings of different studies could be due to the usage of various forms of SO, including sun-dried ground leaves, purified plant extracts, or sage essential oil, that have different mechanisms of action on ruminal fermentation and microorganisms.

The asymptotic GP of CO_2_ and the rate of CO_2_ increased linearly during the study, which contrasted with the findings of Bokharaeian et al. [18] where the CO_2_ decreased with greater levels of sage essential oil supplementation in vitro [18]. Increasing the propionate concentration at the expense of acetate could increase CO_2_ [43]. However, the mechanism behind the increased CO_2_ levels with the increasing level of SO herein is challenging to reconcile since the greater propionate level was observed at 1% SO inclusion but not at the expense of acetate or butyrate, which increased with the propionate. Hence, the decreased use of CO_2_ may be attributed to the inhibition of CH_4_ production by the plant secondary compounds of SO.

### 4.4. Degradability and Fermentation

Evaluating the stability and equilibrium of the rumen environment in ruminant animals requires assessing the ruminal pH, which normally ranges from 5.0 to 7.5 [44]. The pH in the current study changed linearly and quadratically, with the lowest pH observed at 1% inclusion of SO, and then it increased after that. The total SCFA concentration was also changed in a similar pattern, showing the greatest concentration at 1% of SO inclusion. This supports the fact that a lower pH is related to a higher total SCFA concentration [45]. A higher pH with higher doses of sage essential oil was reported by Bokharaeian et al. [18], which agrees with the findings of this study. The molar concentration of acetate changed linearly and quadratically and that of the propionate changed quadratically, with the highest concentration of both SCFAs shown at 1% inclusion of SO and then decreased with higher inclusion. Whereas Zmora et al. [9] showed a linear decrease in propionate concentration with the incremental level of dried sage leaves, Castillejos et al. [46] proposed increased levels of propionate at the expense of acetate and butyrate, which decreased with the supplementation of sage oil (0–500 mg/L) [9,47]. However, butyrate was not changed in the current study with the incremental dose of SO. Collectively, the inclusion of 1% SO increased the concentration of acetate and propionate, suggesting that it enhanced the utilization of carbohydrates in the diet.

Whereas NH_3_-N concentration changed quadratically with 1% showed higher concentration then steadily declined with higher doses, other studies reported a linear increase in ammonia concentrations with increasing levels (0, 250, 500, 750, 1000 mg/L) of sage essential oil [12]. Contrarily, Hosseini et al. [10] and Bokharaeian et al. [18] reported decreased concentrations of NH_3_ or NH_3_-N with higher doses of SO and sage essential oil. Considering the balance between the release of ammonia from protein degradation and its assimilation by rumen bacteria, as indicated by Nolan and Dobos [48], it is plausible to suggest that an increase in ammonia levels could be attributed to a corresponding decrease in microbial biomass at a particular dose of SO supplementation. Elevated NH_3_ production in the rumen is associated with the inefficient utilization of dietary protein and higher nitrogen excretion [49]. We observed the lowest concentration of NH_3_-N with the highest dose of SO which may be caused by the formation of a phenolic–protein complex that reduces the degradability of protein and thus NH_3_ concentrations [10]. The decrease in ammonia levels may also be linked to the bioactive chemicals found in phytogenic feed additives, which could impede the function of ruminal microbial enzymes responsible for producing ammonia. Adding SO at 1% of the diet DM enhanced the ME, PF_24_, and MCP levels, indicating an ideal combination of energy and protein, leading to increased microbial protein synthesis and PF_24_ [50]. The phenolic compounds in SO may interact with biosynthesis of aromatic amino acids, as both synthesis pathways are linked through phytochemicals from SO [51]. Results from increased MCP indicated that most NH_3_-N and SCFA were used for MCP synthesis [52].

The degradability of *d*DM and *d*NDF showed a dose-dependent response, with the higher *d*DM and *d*NDF observed at 1% inclusion which then steadily declined thereafter. It has been demonstrated previously that a greater dose of essential oils from plant extracts may suppress the growth of cellulolytic bacteria in the rumen, potentially decreasing the degradability of feedstuffs [53,54]. Zmora et al. [9] showed that including SO reduced the number of cellulolytic bacteria *Ruminococcus flavefaciens,* and dry matter digestibility decreased with the SO supplementation [12]. However, the greater degradability shown in the 1% inclusion of SO in this the study could be described by the positive influence of volatile compounds on rumen fibrolytic bacterial activity that eventually enhanced the degradation and fermentation of the substrates [12]. Many other studies did not see any effect on *d*DM and *d*NDF with the supplement of sage essential oil [12,17]. The dissimilarities in findings for nutrient degradability among studies are due to the level of doses and the forms of SO supplied (dried ground leaves vs. plant extract of essential oil). These results are certainly promising; however, procedures for harvesting the SO need to be evaluated to formulate a more homogenous product that could overcome further potential challenges for widespread implementation. In addition, further in vivo experiments are needed in different livestock (e.g., sheep, goat, and dairy cattle) to investigate the effectiveness of different forms (dry ground leaves vs. plant extract oil) of SO in nutrient digestibility, ruminal fermentation, and gas production kinetics.

## 5. Conclusions

The findings of this study highlight a dose-response effect of SO and its use as a phytogenic feed additive in animal diets. The inclusion of 1% SO of the total diet DM yielded greater gas production and decreased ruminal CH_4_ production in vitro, which could eventually lead to lower environmental footprints for livestock and promote animal sustainability. Moreover, the study also demonstrated that the total SCFAs, the molar concentration of individual SCFAs (i.e., acetate and propionate), and the degradability of DM and NDF increased with 1% SO supplementation in the diet. Collectively, 1% SO supplementation improved gas production kinetics, rumen digestibility, and fermentation. Further research is needed to investigate the incremental level of SO in vivo and evaluate the production performance and changes in the rumen microbiome of ruminant animals.

## Figures and Tables

**Table 1:** Chemical composition of *Salvia officinalis* shrub and incubated diet (g/kg DM).

	*Salvia officinalis*	CGM ^1^	Berseem Hay	Rice Straw	Diet ^2^
Dry matter	931	903	890	940	893
Organic matter	821	923	884	851	819
Crude protein	56	165	128	42	136
Ether extract	94	47	54	19	62
Nonstructural carbohydrates	306	414	224	166	359
Neutral detergent fiber	365	297	478	624	379
Acid detergent fiber	177	175	381	394	240

^1^ Concentrated grain mixture (CGM): 1 kg of CGM DM consisted of 395 g wheat bran, 170 g soybean meal, 395 g ground corn, 10 g vitamin and mineral mixture, and 10 g salt. ^2^ Diets: 500 g concentrate mixture, 400 g berseem hay, and 100 g rice straw per kg of DM.

**Table 2:** Volatile compounds in *Salvia officinalis* shrub identified by HPLC analysis.

Compound ^1^	Concentration ^2^ (%)
*α*-Pinene	15.85
*α*-Thujone	15.62
Camphor	15.20
1,8-cineole	13.20
*β*-Pinene	10.84
Camphene	6.86
Limonene	5.22
*β*-Thujone	3.90
Borneol	2.55
Borneol	2.30
*β*-Caryophyllene	2.20
*p*-Cymene	2.00
*β*-Myrcene	1.52
*α*-Terpineol	0.76
Thujone	0.50
Verbenol	0.37
L-Fenchone	0.35
Estragole	0.31
Linalool	0.28
Tricyclene	0.17

^1^ Identification based on authentic standards, National Institute of Standards and Technology (NIST) library spectra, and the literature. ^2^ Concentrations based on the total areas of the identified peaks.

**Table 3:** In vitro rumen gas production (GP), methane (CH_4_), carbon dioxide (CO_2_) kinetics, and cumulative gas production after 48 h of incubation as affected by increasing levels of *Salvia officinalis* shrub (%, dry matter basis).

	GP Parameters ^1^				CH_4_ Parameters ^2^				CO_2_ Parameters ^3^		
Level	A	c	Lag		A	c	Lag		A	c	Lag
0	78b	0.076ab	1.55bc		29a	0.035b	1.42c		59b	0.036c	2.47
0.5	108a	0.077a	1.43c		27ab	0.040b	1.56bc		66ab	0.049bc	1.87
1	115a	0.077a	1.33c		26abc	0.048ab	1.62abc		65ab	0.053abc	1.70
1.5	77b	0.076ab	1.89ab		23bc	0.053ab	1.78ab		70a	0.063ab	1.90
2	71b	0.069b	2.08a		22c	0.060a	1.87a		70a	0.074a	2.11
SEM	2.4b	0.0017	0.076		0.8	0.0041	0.057		2.3	0.0049	0.229
*p* value											
Treatment	<0.001	0.022	<0.001		0.003	0.009	0.002		0.035	0.003	0.232
Linear	0.001	0.017	0.001		0.002	0.006	<0.001		0.005	0.002	0.360
Quadratic	<0.001	0.015	0.002		0.902	0.950	0.988		0.279	0.901	0.042

Means in the same column with different letters differ, *p* < 0.05. *p*-value is the observed significance level of the F-test for treatment; SEM = standard error of the mean. ^1^ GP parameters: A is the asymptotic GP (mL/g DM), c is the rate of GP (/h), Lag is the initial delay before GP begins (h). ^2^ Methane (CH_4_) production parameters: A is the asymptotic CH_4_ production (mL/g DM), c is the rate of CH_4_ production (/h), Lag is the initial delay before CH_4_ production begins (h). ^3^ Carbon dioxide (CO_2_) production parameters: A is the asymptotic CO_2_ production (mL/g DM), c is the rate of CO_2_ production (/h), Lag is the initial delay before CO_2_ production begins (h).

**Table 4:** In vitro rumen fermentation profile of diet with increasing levels of *Salvia officinalis* shrub (%, dry matter basis).

	Degradability ^1^				SCFA ^2^						Fermentation ^3^			
Level	*d*DM	*d*NDF	*d*ADF		Total	C_2_	C_3_	C_4_		pH	NH_3_-N	ME	PF_24_	MCP
0	0.473ab	0.439bc	0.412b		23.4c	11.4b	7.9b	4.1		6.27a	10.4	4.68b	7.22a	328a
0.5	0.521b	0.519a	0.444ab		25.0b	12.5ab	8.7ab	3.8		6.27a	11.8	5.00a	5.72b	321b
1	0.582a	0.555a	0.508a		28.5a	14.0a	9.6a	4.9		6.10b	12.7	5.17a	5.98b	368a
1.5	0.481b	0.459b	0.433b		23.1c	11.3bc	7.7b	4.0		6.33a	11.6	4.28c	7.46a	338ab
2	0.429c	0.411c	0.392b		21.2d	9.7c	7.5b	4.0		6.37a	11.4	4.08c	7.49a	303b
SEM	0.0107	0.0095	0.0146		0.29	0.36	0.27	0.28		0.030	0.50	0.06	0.19	8.6
*p* value														
Treatment	<0.001	<0.001	0.002		<0.001	<0.001	0.002	0.121		0.008	0.102	<0.001	<0.001	0.004
Linear	0.004	0.004	0.293		<0.001	0.002	0.069	0.944		0.018	0.326	<0.001	0.003	0.254
Quadratic	<0.001	<0.001	0.004		<0.001	<0.001	0.008	0.212		0.002	0.021	<0.001	0.001	0.002

Means in the same column with different letters differ, *p* < 0.05. *p*-value is the observed significance level of the F-test for treatment; SEM = standard error of the mean. ^1^ dDM is dry matter degradability (g/g incubated), dNDF is neutral detergent fiber degradability (g/g incubated), dADF is acid detergent fiber degradability (g/g incubated). ^2^ SCFA is short chain fatty acids (mmol/g DM), C_2_ is acetate (mmol/g DM), C_3_ is propionate (mmol/g DM), C_4_ is butyrate (mmol/g DM). ^3^ NH_3_-N is ammonia-N (mg/g DM), ME is metabolizable energy (MJ/kg DM), PF_24_ is the partitioning factor at 24 h of incubation (mg degradable DM: mL gas), MCP is microbial CP production (mg/g DM).

## Data Availability

The original contributions presented in the study are included in the article, further inquiries can be directed to the corresponding authors.
